# Examining Treatment Patterns and Real-World Outcomes in Chronic Lymphocytic Leukemia Using Administrative Data in Ontario

**DOI:** 10.3390/curroncol28060408

**Published:** 2021-11-19

**Authors:** Soo Jin Seung, Manjusha Hurry, Shazia Hassan, Ashlie Elnoursi, Krystin A. B. Scheider, Dennis Wagner, Jonathan J. Edwin, Andrew T. W. Aw

**Affiliations:** 1 HOPE Research Centre, Sunnybrook Research Institute, Toronto, ON M4N 3M5, Canada; Shazia.hassan@gmail.com; 2 AstraZeneca Canada, Mississauga, ON L4Y 1M4, Canada; manjusha.hurry@astrazeneca.com (M.H.); ashlie.elnoursi@astrazeneca.com (A.E.); Krystin.Scheider@astrazeneca.com (K.A.B.S.); dennis.wagner83@gmail.com (D.W.); jonathan.edwin@alumni.ubc.ca (J.J.E.); 3 The Ottawa Hospital General Campus, Ottawa, ON K1H 8L6, Canada; aaw@toh.ca

**Keywords:** CLL, cancer, survival, cost, treatment patterns, Canada

## Abstract

Information on the real-world experience of Canadians diagnosed with chronic lymphocytic leukemia (CLL) is limited. This study was conducted to report treatment patterns and outcomes of CLL using Ontario administrative data. A retrospective cohort study was conducted in patients diagnosed with CLL between 1 January 2010 and 31 December 2017 identified in the Ontario Cancer Registry (OCR). Data were accessed using the Institute of Clinical Evaluative Sciences (ICES), which collects various population-level health information. In the Ontario Cancer Registry, 2887 CLL patients receiving treatment and diagnosed between 2010–2017 were identified. Fludarabine, cyclophosphamide and rituximab (FCR) chemoimmunotherapy was most frequently used as a first line, but use declined since ibrutinib and obinutuzumab combinations were funded in 2015. In patients treated with frontline FCR, survival at year one was 89% pre-2015 and 96% post-2015; at year four, survival was 73% and 87%, respectively. Survival in patients treated with frontline chlorambucil was 76% pre-2015 and 75% post-2015 in year 1, and 45% and 56% in year 3. Our analysis shows that, as the treatment landscape for CLL has shifted, use of newer and novel agents as a first line or earlier in the relapsed/refractory setting has resulted in improved survival outcomes.

## 1. Introduction

Chronic lymphocytic leukemia (CLL) is the most common type of adult leukemia in Canada, accounting for about 44% of all leukemias [1]. In 2016/17, approximately 1745 Canadians were diagnosed with CLL, and 611 Canadians died from the disease [2]. CLL mainly affects older patients, with a median age at diagnosis of 71 years. The five-year net survival rate for CLL is 83% [3].

CLL has a unique disease trajectory, as most patients with CLL are often asymptomatic at presentation, with the majority (>80%) currently being diagnosed at an early stage [4,5]. Many of these patients will have an indolent course for years and usually do not require treatment until the onset of symptoms [4,5]. As such, the optimal first line treatment strategy is largely dependent on the individual patient’s characteristics, including age/fitness level, performance status, and the presence of high-risk cytogenetics, as well as patient preference and social factors, such as caregiver stress and ease of transport [6,7].

The therapeutic landscape and available treatment options for CLL are constantly evolving. During 2010–2017 in Ontario, Canada, for first line treatment of fit, younger CLL patients without del(17p) or TP53 mutation, fludarabine, fludarabine, cyclophosphamide and rituximab (FCR) is a reasonable option that is funded in Ontario, particularly in those with mutated immunoglobulin heavy-chain variable region gene (IgHV) in whom long term remission may be achieved [8]. We recognize that there is data demonstrating that frontline ibrutinib in combination with rituximab is well tolerated and associated with an overall survival advantage in this patient population [9], but this is not currently a publicly reimbursed treatment option in our province. However, most CLL patients were ineligible to receive FCR due to advanced age and increased comorbidities, thus chlorambucil in combination with obinutuzumab (C + O) could be used. Newer targeted therapies now available, including Bruton’s tyrosine kinase (BTK) inhibitors, have proven effective most notably in those with high-risk genetic features, including de117p and TP53 mutations [10,11,12,13], or have unmutated IgHV disease [14]. Due to the superior efficacy and improved tolerability of BTK inhibitors over chemoimmunotherapy regimens such as C + O (and venetoclax with obinutuzumab, although not a publicly reimbursed treatment option in Ontario), clinical practice has shifted towards increased utilization of these targeted agents in first line treatment settings [8].

In Canada, ibrutinib was first approved in November 2014, and is funded in Ontario in untreated patients with high-risk disease in 2017 (for patients with del17p/TP53 mutations, and unmutated IgHV), and broadly in patients with at least one prior therapy in 2015 [15]. Acalabrutinib, administered as monotherapy or in combination with obinutuzumab, is a next generation BTK inhibitor recently approved for untreated CLL and for patients with at least one prior therapy [16]. With the recent approval of acalabrutinib, there is the need to better characterize treatment approaches in Canada to support health technology assessments by bodies such as the Canadian Agency for Drugs and Technologies in Health (CADTH) and Institut national d’excellence en santé et services sociaux (INESSS). Despite 5 years of routine use of new agents, such as ibrutinib and obinutuzumab combinations, there is limited data outside of clinical trials on real-world outcomes for Canadian patients treated with new agents. Consequently, we report on the treatment patterns and outcomes associated with CLL using population level administrative datasets in Ontario, Canada.

## 2. Materials and Methods

### 2.1. Study Design

A retrospective cohort study was conducted in patients diagnosed with CLL between 1 January 2010 and 31 December 2017 identified in the Ontario Cancer Registry (OCR) using relevant International Classification of Diseases for Oncology version 3 (ICD-O-3) codes. Patients had a minimum 6-month follow-up from diagnosis, with 31 August 2019 as the last date of follow-up or date of death (whichever came first). Ontario has a population of 14 million residents and provides publicly funded health care services through the Ontario Health Insurance Plan (OHIP).

### 2.2. Patient Population

To be included, patients had to be at least 18 years of age with valid provincial coverage, diagnosed with CLL based on the ICD-O-3 histology code 9823/3-CLL or small cell leukemia, and survived more than 2 weeks after diagnosis. Patients were included if any of the following treatments were administered regardless of line of therapy: bendamustine, bendamustine + rituximab (BR), chlorambucil, C + O, cyclophosphamide, FCR, ibrutinib, idelalisib + rituximab, rituximab, and venetoclax (venetoclax combination treatment was not funded and thus excluded). Baseline characteristics for treated patients were included.

### 2.3. Data Sources

Data were accessed using the Institute of Clinical Evaluative Sciences (ICES), which collects data on public coverage via the Ontario Health Insurance Plan (OHIP) and other population-level health information in order to generate real-world data. To determine the trajectory of care over time of a patient cohort, health information on each individual patient was linked to applicable datasets. For patients with CLL, linkages were made to a number of datasets to examine treatment patterns and outcomes. Three databases were used to report treatments received by the study cohort. The Ontario Drug Benefit Claims (ODB) database, captured all oral medications and a wide range of supportive care drugs (e.g., analgesics and antiemetics) in patients aged ≥65 years or patients on social assistance. The New Drug Funding Program (NDFP) database was used to capture intravenous systemic chemotherapy agents that are publicly funded by Cancer Care Ontario (CCO). The Activity Level Reporting (ALR) captured treatment information not available in the ODB and NDFP. The Registered Persons Database (RPDB) contains demographic information on all individuals with OHIP coverage (e.g., date of birth, date of death), and was used in determining survival.

### 2.4. Statistical and Costing Analysis

Statistical analyses were performed in SAS Enterprise Guide 7.1. Baseline characteristics were stratified by treatment received in first line, and were summarized by number and percentage for categorical variables and by mean and standard deviation for continuous variables. Treatment patterns were reported as first line treatment and onwards, and were characterized by the number and percentage of patients receiving different types of treatments. First line treatment was defined as the first CLL treatment received after diagnosis, second line treatment was defined as the start of the next CLL treatment after the end of first line treatment, and third line treatment was defined as the start of the next CLL treatment after the end of the second line treatment. Patients who were on concurrent lines of therapy for CLL were excluded.

The primary clinical outcome of interest was overall survival (OS) with mean (including standard deviation (SD)), median, 95% confidence interval and interquartile range (IQR) evaluated using the Kaplan–Meier methods for censored data, and was based on stratification and log-rank test. OS was defined from the time of diagnosis to death by any cause, or last date of follow-up. OS was also assessed from time of treatment initiation stratified by type and line of treatment, to account for delay in start of treatment. Patients were then followed until death or last recorded date of follow-up. As a proxy for ‘progression-free survival’ (PFS) the following outcomes were assessed: ‘PFS proxy in first line (PFSP1)’ was estimated from start of first line treatment to start of second line treatment and ‘PFS proxy in second line (PFSP2)’ was estimated from start of second line treatment to start of third line treatment. For statistical analyses, follow-up data was included until 31 August 2019.

## 3. Results

### 3.1. Baseline Characteristics

In total, 10,008 patients were diagnosed with CLL from 2010–2017 in Ontario, Canada. Of the treated patients, the average age at diagnosis was 68.3 ± 11.3 (median, 69 (61–77)) and 66.6% of patients were male. The average time from diagnosis to start of treatment was 1.78 years (±1.95).

Table 1 reports the distribution of treatment regimens, irrespective of line of therapy. FCR was the therapy most often used in the overall cohort (35%). Use of FCR steadily decreased over the time period studied, with C + O or ibrutinib being more frequently used in 2017 or 2018, and more frequently used in older patients. Patients who received FCR also had less comorbidities at diagnosis compared to patients who received C + O or ibrutinib (Table 1).

### 3.2. Treatment Patterns by Line of Therapy

#### 3.2.1. First Line Treatment

**Figure 1:**
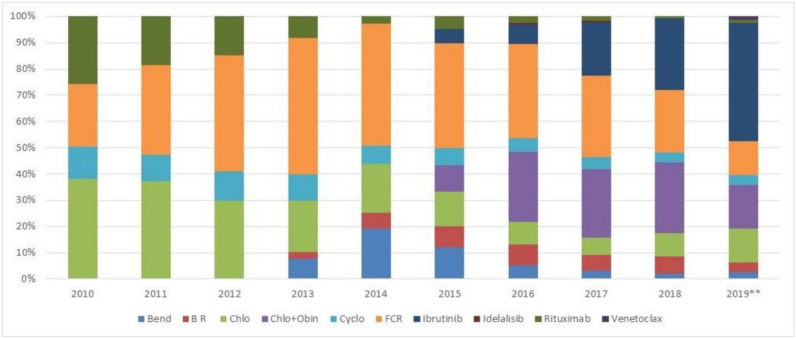
First line treatment regimen received over the years. (Bend: bendamustine; B R: bendamustine + rituximab; Chlo: chlorambucil; Chlo + Obin: chlorambu-cil + obinutuzumab; Cyclo: cyclophosphamide; FCR: fludarabine, cyclophosphamide and rituximab. 2019**: Data available until August 2019).

Figure 1 shows the shift in treatment approach over time. Key changes can be noted as of 2015, at the time of reimbursement of new agents such obinutuzumab (in combination with chlorambucil), and approval and funding of ibrutinib [9,17,18]. As of 2015, treatment approach shifts showed an increase in the use of C + O and ibrutinib, with decreased use of FCR and chlorambucil monotherapy. By 2018, 27% of patients received C + O, 27% received ibrutinib and 24% received FCR. Data for 2019 was available until August 2019 and may omit certain treatment combinations; however, ibrutinib was administered in 45% of patients.

#### 3.2.2. Second Line Treatment

In subsequent lines of treatment, 71% in the overall cohort did not receive any treatment during the follow-up period. For patients who received FCR and who were deemed fit, 68% of patients did not receive subsequent treatment.

**Figure A1:**
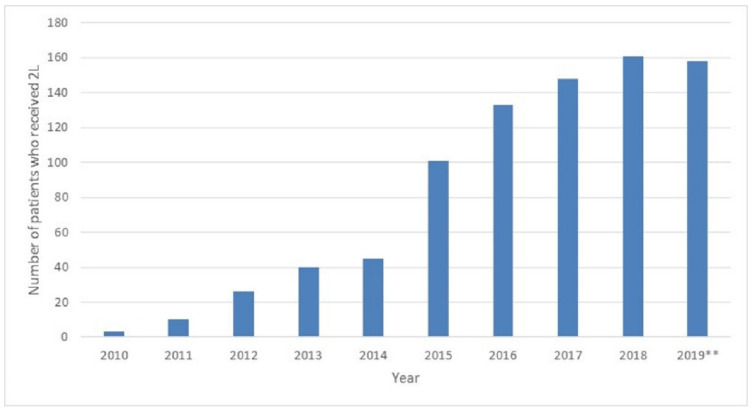
Number of patients who received second line treatment during study period. 2019**: Data available until August 2019.

**Figure A2:**
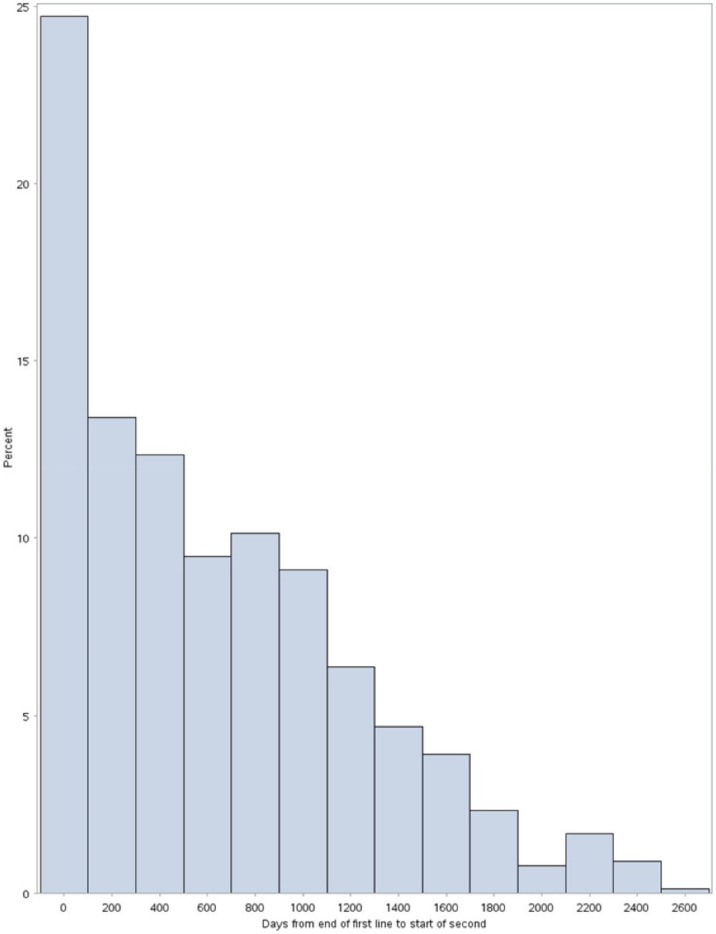
Distribution of time from end of first line treatment to start on second line treatment.

There was an increasing trend towards the administration of second line treatment over the years; by 2015, the number of patients treated doubled compared to 2014 (Figure A1). On average, there was a period of 1.74 years (±1.59) from the end of first line treatment to the start of second line treatment (Figure A2). In patients who were subsequently treated, ibrutinib was the most frequently administered treatment in second line. Of the 2887 patients treated in first line, 827 patients (28%) received a second line treatment, and 65% (534/827) of these patients received ibrutinib. In patients who received FCR in first line and treated in second line, 78% received ibrutinib. In patients receiving front-line C + O (*N* = 421), 128 patients were subsequently treated in second line, of whom 116 (91%) received ibrutinib. In patients treated with front-line ibrutinib (*n* = 352), 10% of patients subsequently progressed to a second line treatment, where 41% received venetoclax and 19% received chlorambucil.

Figure A1 presents the number of patients who received second line treatments stratified by year during the study period.

#### 3.2.3. Third Line Treatment

Similar to second line, the majority of patients (85%) did not progress or switch treatments to receive a third line treatment. One hundred and twenty-four patients received a third line treatment with an average wait of 0.98 years (±1.3). In the overall cohort, 54% received ibrutinib, while 15% received a cyclophosphamide-based regimen regardless of prior treatment. Of the 17 patients who received venetoclax as a second line treatment, upon progression or treatment switch, patients either received ibrutinib (33%), chlorambucil (33%) or cyclophosphamide (33%). In patients who received prior ibrutinib, 41% received venetoclax as a third line.

### 3.3. Survival

**Figure 2:**
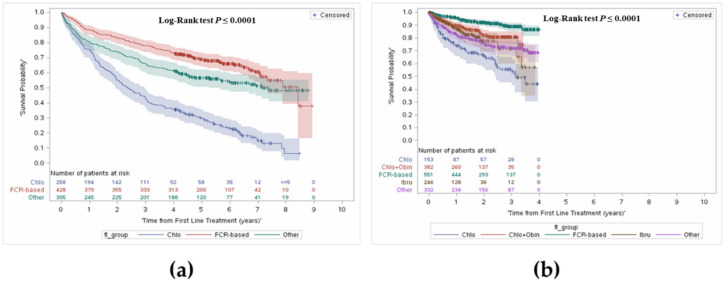
Overall survival from first line treatment stratified by before and after 2015, and by type of treatment. (**a**) Before 2015; (**b**) After 2015.

Survival outcomes included OS, survival from initiation of first line, second line and third line treatments, and landmark survival at 1, 2, 3, 4 and 5 years. To avoid bias due to delay in treatment initiation, OS was assessed from time of treatment initiation. In the treated cohort, median OS was 7.0 years (95% CI: 6.4–7.8). OS was further stratified to capture changes in survival with the public reimbursement of agents such as obinutuzumab combinations or ibrutinib as of 2015 in Ontario [17,18]. Median OS was 6.2 years (95% CI: 5.4–6.9) in patients diagnosed and treated before 2015, and median OS was not reached in patients who were diagnosed and initiated treatment after 2015 (Table 2, Figure 2), as patients diagnosed before 2015 had a longer follow-up period, compared with patients diagnosed after 2015, who would have had a maximum follow-up of 4.5 years.

Comparing landmark survival rates in pre-2015 to post-2015, an increase in survival can be noted, most notably in the FCR group, with an average increase of 10%. Improvement can be observed in the chlorambucil group, with an increase of 11% in year 2 and year 3.

#### 3.3.1. Overall Survival from First Line Treatment Initiation

**Figure 3:**
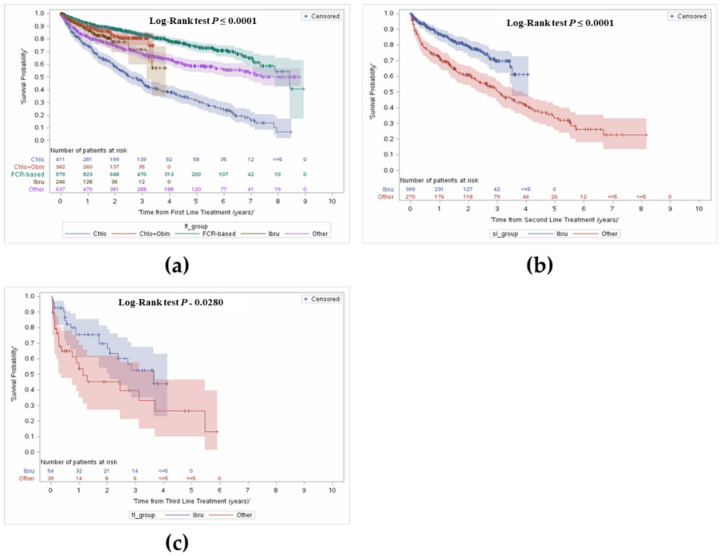
Overall survival from first, second and third line treatment stratified by type of treatment received. (**a**) Overall survival from first line treatment; (**b**) Overall survival from second line treatment; (**c**) Overall survival from third line treatment.

OS was further stratified from time of treatment initiation and by treatment received by line of therapy. The median follow-up was 3.9 years (interquartile range: 2.2–5.9). Figure 3 presents OS by line of treatment. In the overall cohort, survival from first line treatment at 1 year was 86% and 61% at 5 years.

In the FCR group, 93% of patients were alive at year 1, 83% alive at year 3, and a majority remained alive at year 5 (74%) (median OS = 7.0 years). In patients who received C + O, survival at year 1 was 90%, with 81% alive at year 3. Survival was lowest in the chlorambucil monotherapy group. Survival in the ibrutinib group was higher than the chlorambucil monotherapy group at year 1 (87% vs. 75%), and at year 3 (72% vs. 48%).

#### 3.3.2. Overall Survival from Initiation of Second or Third Line Treatment

Figure 3 shows OS from initiation of second and third line treatments. Given only 29% of patients received a second line treatment, treatment was stratified by ibrutinib use as it made up the majority of treatment compared to the ‘other’ group. Median OS in the overall cohort was 4.0 years (95% CI: 3.5–4.9). Median OS was 3.2 years in the ‘other’ group and was not reached in the ibrutinib group. Survival with ibrutinib was consistently longer compared to the ‘other’ group; 87% vs. 73% in year 1, and 61% vs. 42% in year 4, respectively.

Similarly, OS was explored from initiation of third line treatment onwards. Median OS in the overall cohort was 2.7 years (95% CI: 1.7–5.5), and longer in patients who received ibrutinib compared to the ‘other’ group (3.8 vs. 1.3 years). Survival over time was consistently longer in the ibrutinib group; survival at year 1 was 75% compared to 57% in the other group, and 70% vs. 45% in year 2.

### 3.4. Other Outcomes: Time to Subsequent Treatments

**Figure 4:**
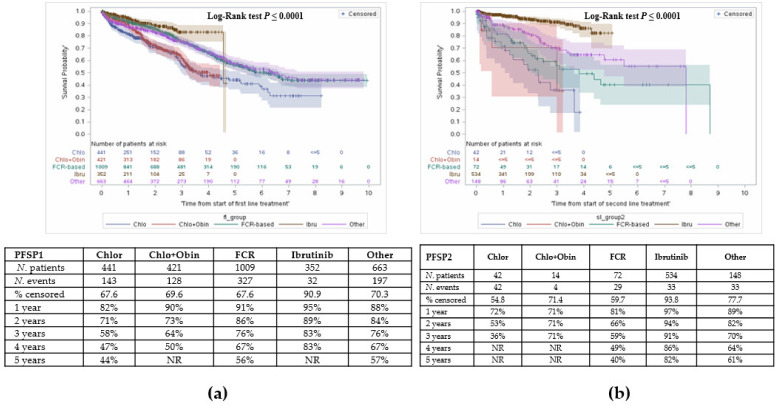
Time between treatments. (**a**) Time from initiation of first line treatment to second line treatment; (**b**) Time from initiation of second line treatment to third line treatment.

At 1 year, PFSP1 was highest with ibrutinib and lowest with chlorambucil. At year 4, PFSP1 was comparable between C + O or chlorambucil monotherapy (Figure 4a).

Time from start of second line treatment to start of third line treatment was also assessed as a proxy for PFS (Figure 4b). Patients who received ibrutinib remained ‘progression-free’ the longest, compared to other agents.

Figure A2 presents the proportion of patients per time frame of 200 days between time from end of first line treatment to start of second line treatment

## 4. Discussion

There are limited studies characterizing real-world treatment patterns in patients with CLL in Canada. The objective of this study was to describe changes in treatment approach over eight years and assess changes in survival in patients diagnosed and treated with CLL in the province of Ontario.

Our study describes the shift in treatment approach with the public funding of novel agents [9,17,18]. Over the study period, chemoimmunotherapy was most commonly used, however there has been a steady decrease in FCR use since 2015 with the public funding of obinutuzumab combinations in the first line setting and since 2017 with the funding of ibrutinib in the first line setting. Even though the majority of patients did not receive a second line treatment in this cohort, treatment upon progression from first line appeared to increase, likely reflecting access to ibrutinib with public funding in the relapsed/refractory (R/R) setting [18]. Reasons for the high proportion of patients who did not receive treatment beyond frontline may include: (1) the follow-up is not substantially long enough to observe a treatment switch; (2) some patients may not have needed a subsequent line of treatment as they were in long-term remission, and thus did not experience recurrence requiring treatment; (3) some patients may have died before receiving a subsequent line of treatment.

Assessment of survival in pre and post 2015, suggests an improvement in overall survival. The results of our study corroborate the findings of a similar study in British Columbia [19]. Of 1729 patients included in the study, with a median age at diagnosis (66 years) slightly younger than our cohort (71 years), FCR was the most frequently used during the study period (1984–2016), with an uptake of ibrutinib in recent years [19]. The authors demonstrate an improvement in survival in patients treated with ibrutinib during recent time periods (median OS was not reached in patients treated between 2014–2016 vs. 11.9 years in patients treated between 1984–2014, *p* < 0.001) [19].

In our study, an increase in landmark survival rates pre-2015 vs. post-2015 was noted in the FCR (average increase 10%) and chlorambucil groups (11% increase years 2 and 3). Some patients who were deemed to be candidates only for chlorambucil monotherapy pre-2015 would have been fit enough to receive C + O after July 2015 when the combination therapy was funded, which may partly explain the improvement in survival in the chlorambucil group.

As ibrutinib use in first line is restricted to a high-risk population (defined as the presence of chromosome 17p deletion, or TP53 mutation or unmutated IgHV), the OS reported in this study likely reflects the poor prognosis of this group [20]. Deletion 17p has also been associated with high risk of disease progression and poor survival [21], with a median OS of 2 to 3 years from time of first line treatment initiation [22]. It is unclear whether unmutated IgHV is a poor prognostic factor when patients are treated with ibrutinib. In a recently pooled study of ibrutinib trials, it was demonstrated that 93% of patients with unmutated IgHV who received ibrutinib were alive at year 1, 88% at year 2, and 83% at year 3 [23]. Survival in the untreated population was 86% at year 1, 77% at year 2, 70% at year 3 and 63% at year 4 [23]. Although this represents a high-risk population, the outcomes with ibrutinib are still likely significantly improved compared to what would have been expected with conventional chemoimmunotherapy. While other ibrutinib real-world studies have been published [24,25], our study is the largest Canadian study using provincial administrative data of CLL patients treated with publicly reimbursed therapies, which includes ibrutinib.

In patients who received C + O, survival was lower than those who received FCR (Figure 3). The lower survival in the C + O group is likely due to the fact that it is usually administered in patients who are unfit to tolerate FCR (e.g., older patients or those who have comorbidities such as renal impairment) [19]. Survival was lowest in the chlorambucil monotherapy group, likely a reflection of older age, unfit status and minimal duration of response expected from chlorambucil monotherapy. Survival among patients who received ibrutinib in our cohort was lower than FCR or C + O and can be explained in part by the use of ibrutinib in a high-risk (older and less fit) group in the R/R setting. In our cohort, patients who discontinued or progressed on ibrutinib as a first line and were subsequently treated, received chlorambucil most frequently, suggesting they were not fit for anything else, until funding of venetoclax became available. Among patients who progressed on other agents administered in first line, ibrutinib was the most frequently used treatment upon progression. This can be further explained by comparing the ‘progression-free survival proxy’ period across agents whereby patients who received ibrutinib appear to have a better outcome compared to other agents.

Nonetheless, interpretation of survival may be limited by a number of factors: (1) administrative data does not capture CLL prognostic markers, safety outcomes or dose modifications for ibrutinib; (2) stage at diagnosis of disease; (3) performance status of patients; (4) heavy censoring; (5) this analysis only includes publicly funded therapies, so would not have included therapies during clinical trial or through compassionate supply (i.e., a number of patients who progressed on ibrutinib would have gone on to compassionate supply of venetoclax before public reimbursement was available, and this would not have been captured); (6) public funding of oral agents is for Ontarians 65+ years or on social assistance (regardless of age) so oral agent utilization of people < 65 years is very limited; (7) this study did not explore the use of allogeneic stem cell transplant and potentially underestimated subsequent treatments received. Due to heavy censoring in the progression free survival analysis these results should be interpreted with caution.

## 5. Conclusions

Our analysis shows that novel agents such as ibrutinib and other small molecule inhibitors have replaced chemoimmunotherapy use in the front line and R/R setting. The change in treatment approach resulted in rapid improvement in survival outcomes. There is room for improvement with approved next generation BTK inhibitors such as acalabrutinib, which has improved selectivity and BTK occupancy. Future research should continue to track patient outcomes with the introduction of additional novel agents in Canada, and further analysis is required to estimate the predicted costs of treating CLL over a patient’s lifetime to account for new and novel therapies, and to ultimately aid in funding decisions.

## Figures and Tables

**Table 1:** Demographic table of treated patients diagnosed with CLL in Ontario between January 2010 to December 2017 stratified by treatment regimen received.

	C + O *N* = 421	FCR-Based *N* = 1009	Ibrutinib *N* = 352	Other * N = 1105	Total *N* = 2887
**CLL Diagnosis Year**					
2010	28 (6.7%)	119 (11.8%)	10 (2.8%)	185 (16.7%)	342 (11.8%)
2011	27 (6.4%)	137 (13.6%)	22 (6.3%)	172 (15.6%)	358 (12.4%)
2012	32 (7.6%)	145 (14.4%)	17 (4.8%)	145 (13.1%)	339 (11.7%)
2013	45 (10.7%)	151 (15.0%)	22 (6.3%)	170 (15.4%)	388 (13.4%)
2014	54 (12.8%)	148 (14.7%)	39 (11.1%)	125 (11.3%)	366 (12.7%)
2015	91 (21.6%)	125 (12.4%)	68 (19.3%)	120 (10.9%)	404 (14.0%)
2016	72 (17.1%)	89 (8.8%)	76 (21.6%)	101 (9.1%)	338 (11.7%)
2017	72 (17.1%)	95 (9.4%)	98 (27.8%)	87 (7.9%)	352 (12.2%)
**Age at Diagnosis**					
Mean ± SD	73.55 ± 6.84	61.09 ± 9.39	67.92 ± 10.67	73.09 ± 10.82	68.33 ± 11.28
Median (IQR)	74 (69–78)	61 (55–67)	69 (62–76)	74 (66–82)	69 (61–77)
**Age at Treatment**					
Mean ± SD	76.09 ± 6.38	62.63 ± 9.17	70.60 ± 10.33	74.47 ± 10.77	70.10 ± 11.17
Median (IQR)	76 (72–81)	63 (56–69)	71 (65–77)	76 (67–83)	71 (63–78)
**Sex**					
Female	131 (31.1%)	299 (29.6%)	105 (29.8%)	430 (38.9%)	965 (33.4%)
Male	290 (68.9%)	710 (70.4%)	247 (70.2%)	675 (61.1%)	1922 (66.6%)
**Income Quintile**					
Missing	1–5 **	1–5 **	1–5 **	1–5 **	9 (0.3%)
1 (Lowest)	80 (19.0%)	155 (15.4%)	47 (13.4%)	204 (18.5%)	486 (16.8%)
2	76–80 **	202–206 **	70–74 **	245–249 **	604 (20.9%)
3	89 (21.1%)	187 (18.5%)	73 (20.7%)	198 (17.9%)	547 (18.9%)
4	87 (20.7%)	221 (21.9%)	75 (21.3%)	231 (20.9%)	614 (21.3%)
5 (Highest)	84 (20.0%)	239 (23.7%)	82 (23.3%)	222 (20.1%)	627 (21.7%)
**Charlson Comorbidity Index**					
Mean ± SD	1.06 ± 1.65	1.30 ± 1.59	0.95 ± 1.47	1.92 ± 2.03	1.46 ± 1.81
Median (IQR)	0 (0–2)	0 (0–2)	0 (0–2)	2 (0–3)	1 (0–2)
**Comorbidities**					
Chronic obstructive pulmonary disease (COPD)	85 (20.2%)	131 (13.0%)	65 (18.5%)	250 (22.6%)	531 (18.4%)
Diabetes	128 (30.4%)	188 (18.6%)	90 (25.6%)	294 (26.6%)	700 (24.2%)
Myocardial infarction (MI)	18 (4.3%)	34 (3.4%)	14 (4.0%)	48 (4.3%)	114 (3.9%)
Congestive heart failure (CHF)	38 (9.0%)	31 (3.1%)	20 (5.7%)	100 (9.0%)	189 (6.5%)
Rheumatoid arthritis (RA)	9–13 **	12 (1.2%)	1–5 **	20 (1.8%)	46 (1.6%)
Prior cancer	82 (19.5%)	96 (9.5%)	66 (18.8%)	232 (21.0%)	476 (16.5%)

* Other treatments/regimens include bendamustine (monotherapy or in combination with rituximab), cyclophosphamide, rituximab monotherapy, chlorambucil monotherapy, idelalisib and venetoclax. ** Exact counts suppressed due to small cell size and to prevent back calculations.

**Table 2:** Survival rates stratified by type of treatment received in first line before and after 2015.

Before 2015						
	**Overall**	**FCR**	**Chlo + Obin**	**Ibrutinib**	**Chlorambucil**	**Other**
**Median (Years)**	6.2	8.6			2.4	7.3
**Landmark Survival by Year**						
1		89%	NA	NA	76%	81%
2		83%			56%	74%
3		78%			45%	66%
4		73%			36%	62%
5		69%			30%	56%
**After 2015**						
	**Overall**	**FCR**	**Chlo + Obin**	**Ibrutinib**	**Chlorambucil**	**Other**
**Median (Years)**	NR	NR	NR	NR	3.3	NR
**Landmark Survival by Year**						
1		96%	90%	87%	75%	84%
2		92%	82%	78%	66%	76%
3		89%	81%	72%	56%	73%
4		87%	NR	NR	NR	69%

## Data Availability

The data presented in this study are available on request from the corresponding author.
